# Equal levels of pre- and postsynaptic potentiation produce unequal outcomes

**DOI:** 10.1098/rstb.2023.0235

**Published:** 2024-07-29

**Authors:** Leonid P. Savtchenko, Dmitri A. Rusakov

**Affiliations:** ^1^ UCL Queen Square Institute of Neurology, University College London, London WC1N 3BG, UK

**Keywords:** long-term potentiation, realistic cell model, stochastic synapses, presynaptic potentiation, postsynaptic potentiation, input–output function

## Abstract

Which proportion of the long-term potentiation (LTP) expressed in the bulk of excitatory synapses is postsynaptic and which presynaptic remains debatable. To understand better the possible impact of either LTP form, we explored a realistic model of a CA1 pyramidal cell equipped with known membrane mechanisms and multiple, stochastic excitatory axo-spinous synapses. Our simulations were designed to establish an input–output transfer function, the dependence between the frequency of presynaptic action potentials triggering probabilistic synaptic discharges and the average frequency of postsynaptic spiking. We found that, within the typical physiological range, potentiation of the postsynaptic current results in a greater overall output than an equivalent increase in presynaptic release probability. This difference grows stronger at lower input frequencies and lower release probabilities. Simulations with a non-hierarchical circular network of principal neurons indicated that equal increases in either synaptic fidelity or synaptic strength of individual connections also produce distinct changes in network activity, although the network phenomenology is likely to be complex. These observations should help to interpret the machinery of LTP phenomena documented *in situ*.

This article is part of a discussion meeting issue ‘Long-term potentiation: 50 years on’.

## 1. Introduction

The principle of memory trace formation proposed by Hebb refers to the strengthening of a synaptic connection upon the spatiotemporal coincidence of excitatory inputs converging onto that connection [1]. This concept has found its most prominent experimental corroboration in the long-term potentiation (LTP) of excitatory transmission [2,3]. The majority of excitatory connections in the mammalian cortex operate by releasing the neurotransmitter glutamate from presynaptic axonal specializations, a process that ultimately underpins rapid information processing and storage by neural circuits. Following decades of debate, a large body of experimental evidence has argued that the prevailing cellular mechanism of LTP at ‘common’ cortical synapses relies on an increased current through postsynaptic the α-amino-3-hydroxy-5-methyl-4-isoxazolepropionic (AMPA) receptors [4]. Historic experimental evidence for the LTP-associated increase in glutamate release probability [5,6] has subsequently been countered by the silent-synapse hypothesis [7,8] and by reports that detected no increases in the post-stimulus astroglial glutamate uptake after LTP induction [9,10]. However, the LTP-associated boost of release probability at non-silent synapses was later documented [11–15], including genetic [16] and ultrastructural [17] evidence for facilitated synaptic vesicle release post-induction. Theoretically, an increase in the postsynaptic current would require an increased surface density of AMPA receptors, rather than simply a numeric expansion of their pool [18,19], whereas the latter would potentiate the current if new presynaptic release sites appear post-LTP [17]. Thus, a ‘reconciliatory’ view has emerged [20–22], pointing to the distinct involvement of pre- and postsynaptic components of LTP depending on the potentiation-inducing neural activity and the circuitry involved [15,23,24].

Importantly, attempts to relate LTP to a memory formation task *in vivo* have not yet reached single-synapse resolution, which would appear essential for understanding the population dynamics of synaptic strengths and release probabilities during activity-dependent plasticity events [25–29]. The breakthrough dual-eGRASP labelling technique has provided an important step in this direction, by identifying populations of hippocampal connections that, at least according to several indirect indicators, displayed pre- and postsynaptic potentiation following a memory task [30].

While the comparative importance of pre- and postsynaptic L TP mechanisms thus remains debatable and is likely to depend on the brain scenario at hand, one routine assumption has been that equivalent increases in the postsynaptic current and in presynaptic release probability *P*_r_ have a similar outcome for the postsynaptic excitation when averaged over time and across the population of synaptic inputs. However, the probabilistic nature of synaptic discharges and the nonlinearity of dendritic integration mechanisms suggest that this may not necessarily be the case: synaptic events at the extremes of the probability spectrum may produce a postsynaptic response that is at variance with the expected average. The present study was therefore an attempt to explore these relationships using a realistic biophysical model of a well-rehearsed CA1 pyramidal cell equipped with known membrane mechanisms [19,31,32] and a population of stochastic excitatory synapses [33].

## 2. Methods

### (a) CA1 pyramidal cell microcircuit

**Figure 1.**
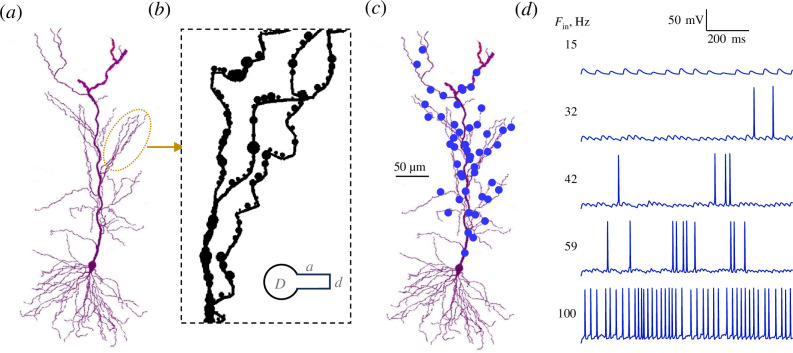
Exploring the effects of pre- and postsynaptic LTP on signal transfer in the CA3–CA1 circuitry in silico. (*a*) Diagram of a reconstructed CA1 pyramidal cell diagram in the NEURON simulation environment [32,35] (see §2 for detail). (*b*) Diagram depicting apical cell dendrites populated stochastically with dendritic spines whose morphological features (neck diameter *d*, neck length *a*, head diameter *D*as indicated) are distributed in accordance with the STED microscopy data [37] (note that NEURON's shape rendering masks spine necks). (*c*) An illustration of the pattern of 50 stochastic excitatory synapses (blue dots) scattered among apical dendrites of the modelled cell (see §2 for synaptic parameters). (*d*) An example of the modelled cell firing in response to different frequencies of presynaptic axonal firing, as indicated. Simulations generated by the current version of the BRAINCELL platform (https://github.com/Rusakovlab/BrainCell).

We explored a realistic NEURON-compatible [34] model of a hippocampal CA1 pyramidal neuron reconstructed morphologically in an acute slice experiment and equipped with the known experiment-derived membrane mechanisms (https://modeldb.science/2796) [32,35]. Simulations were run with a variable time step *dt*; the cell axial specific resistance was set at *R_a_* = 90 Om·cm, as established earlier by fitting *R_a_* to a voltage clamp error (in the readout of the excitatoty postsynaptic current (EPSC)) arising under partial antagonism of AMPA receptors [19], capacitance at *C_m_* = 1 mF cm^–2^, temperature at 34°C, throughout simulations. The apical dendrites were populated stochastically with dendritic spines that possess distributed morphological properties set using the simulated-morphogenesis method demonstrated previously for nanoscopic astrocyte processes [36] and implemented in the current version of our simulation platform BRAINCELL (https://github.com/Rusakovlab/BrainCell). The key structural parameters of the spines (neck length, neck diameter and head diameter) were generated to match the corresponding experimental distributions obtained with super-resolution stimulated emission depletion (STED) microscopy for CA1 pyramidal cells [37] (figure 1*a,b*). We have developed the specialized NEURON-integrated module _SynEventsFilterWatcher.mod to control and analyse the probability of neurotransmitter release (https://github.com/Rusakovlab/BrainCell).

Fifty excitatory axo-spinous synapses were thus distributed over the spines of apical dendrites, at a distance ranging between 10 and 350 µm from the soma (figure 1*c*). The synaptic conductance *G_s_(t*) was modelled using the NEURON function *Exp2Syn*, where *τ*_1_ = 0.1 ms and *τ*_2_ = 10 ms are the rise and decay time constants, respectively, and *G_m_* is the peak conductance factor (under the present *τ*_1_ and *τ*_2_ values, *G_m_* = 0.94 of the actual peak); the *G_m_* value was varied between 0.4 and 0.8 nS, within its physiological range for CA3–CA1 synaptic connections [38]. The reverse potential of excitatory synapses was 0, to reflect the stoichiometry of AMPA and *N*-methyl-d-aspartate (NMDA) receptor types. Each stimulation run lasted for 1000 ms, commencing at a membrane potential of −80 mV, with a delay of 10 ms after achieving full model stabilization. The count of action potentials was calculated over a duration of 1000 ms.

### (b) Network of pyramidal neurons

To monitor basic features of the neural network activity changes caused by synaptic potentiation, we explored a non-hierarchical pyramidal-neuron network, with circular connectivity as described previously [39,40]. The choice of circular connectivity was prompted by several advantages, such as eliminating edge effects (present in networks with non-symmetrical boundaries), equal contribution of all cells and the network size defined by a single parameter, network radius *R*.

We used general settings of a previously published model [41], which was further developed as a full-scale cloud-computing platform ARACHNE [42], with minor modifications of the internal parameters. The network consisted of 200 pyramidal cells. Each cell was modelled as a single compartment using standard Hodgkin–Huxley formalism. The kinetics of synaptic conductance in individual cells was as shown above, with *G_m_* density set at 0.14 µS cm^–2^ (surface area and *G_m_* density being single-compartment parameters).

In the network, the pattern of synaptic connections, rather than being evenly random, followed the empirically derived concept of (quasi-) clustered cortical connectivity [43,44], so that the connection strength matrix *W*(*i,j*) reflected the Gaussian distribution of connections centred at a given neuron, in accord with:


$$W(i,j)=W(i,j{)}_{max}\;exp\left(-\frac{1}{2}{\left(\frac{max(i-j)-1}{\sigma }\right)}^{2}{\left(\frac{j-1}{max(j)}-\frac{j-1}{max(i)}\right)}^{2}\right),$$

where *W*_max_=1 and spatial dispersion *σ* =12. The size (radius) of the network was set at 200 μm, with the signal propagation speed at 0.1 μm ms^−1^, to reflect the basic properties of local networks in the rodent cortex. The network was activated with a 1 kHz stochastic depolarization noise with a conductance density of 0.005 mS cm^–2^ [41].

Simulations were performed using the ARACHNE platform [42] on the Amazon AWS cloud computing (cluster c5.large, tolerance 10^5^, time step 0.02 ms). Random generator *use32BitRng* (MATLAB) was set to generate a delta-correlated white noise for any stochastic processes. To avoid full network synchronization (as opposed to the more realistic attractor mode), the initial resting cell membrane potential was set uniformly randomly, between −73 and −67 mV; the release probability *P*_*r*_ was set at either 0.2 or 0.3, as explained.

The mean firing frequency parameter *f_m_* was calculated as an average of all frequencies for each neuron. We note that the parameter *f_m_* is not the same as the main network frequency, which is computed as a frequency for the maximum power spectrum density function. In other words, the mean firing frequency parameter does not necessarily reflect the dominant oscillation frequency of the network.

The network synchronization parameter *k*(*τ*) was calculated as an average of all coefficients *k_i,j_* (*τ*) for each pair of neurons (*i, j*). The time window of synchronization was divided into bins so that *τ* = 0.1*f_m_* ^−1^ . In each bin, an action potential was represented in a binary format (yes–no series). Finally, *k_i,j_* (*τ*) was calculated for each pair of neurons for the time bin *τ* using the formula:


$$k_{i,j}=\frac{\sum_{m=1}^{K}X_{i}(m)Y_{i}(m)}{{\left(\sum_{m=1}^{K}X_{i}(m)\sum_{m=1}^{K}Y_{i}(m)\right)}^{\frac{1}{2}}},$$

where *X_i_* and *Y_j_* are the binary series of the *i*th and *j*th cells, respectively, and *m* is the bin number; thus, *X*(*m*) and *Y*(*m*) take values of either 0 or 1 depending on having a spike (1) or no spike (0) event, for the (*i,j*) connection during the *m*th bin, with *K* being the total number of bins.

## 3. Results

### (a) Signal transfer function under equal pre- and postsynaptic potentiation

First, we simulated CA1 pyramidal cell activity driven by 50 stochastic excitatory synapses (figure 1*c*) activated by a regular train of axonal action potentials at different frequencies. The aim was to evaluate theoretically the dependence between the input intensity, which is represented by the frequency of afferent firing, and the postsynaptic output (average cell firing frequency). For the control condition, we set the release probability *P*_*r*_ at 0.2, and synaptic conductance *G*_*m*_ at 0.66 nS, in line with the experiment-based estimates for CA3–CA1 circuitry [33,38,45]. In these settings, increasing the input frequency led to a steady increase in the postsynaptic firing intensity, as expected (example in figure 1*d*).

Next, we compared two basic synaptic potentiation scenarios. The first scenario was to increase release probability *P*_*r*_ by 50% (a mid-range synaptic strength increase for the classical LTP), from 0.2 to 0.3 while keeping the synaptic conductance *G*_*m*_ unchanged. In the second scenario, the roles were reversed: *G*_*m*_ was to increase by 50% to 1 nS whereas *P*_*r*_ remained unchanged at 0.2. In 10 trials for each of these scenarios (including control settings), we collected the postsynaptic cell firing data under the presynaptic axon firing frequency ranging between ~10 and 100 Hz.

**Figure 2.**
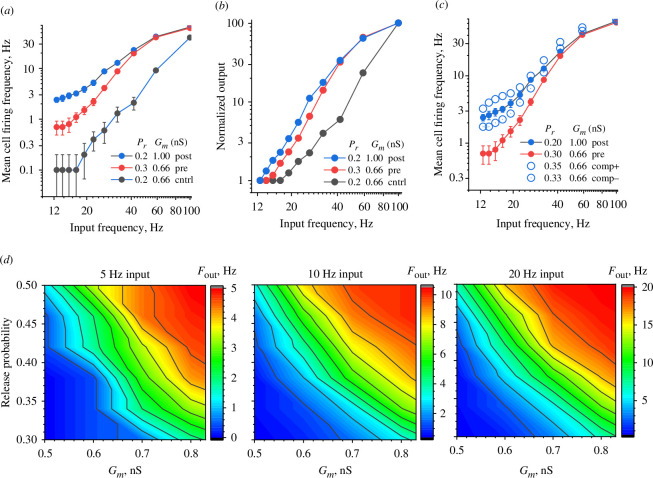
Postsynaptic potentiation mechanism generates a greater effect on cell firing than presynaptic. (*a*) The frequency of postsynaptic cell firing plotted against the presynaptic afferent firing frequency, for the control conditions (cntrl) and two potentiation scenarios (pre and post), as indicated, in the simulated CA3–CA1 circuitry; dots, mean ± s.e.m., *n* = 10 trials; log–log scale. (*b*) The input–output dependencies are as in (*a*), but normalized to the output range of 1–100; log–log scale. (*c*) The firing output under 50% postsynaptic (*G_m_*) potentiation could be achieved with a 65%–75% increase of presynaptic (*P*_*r*_) potentiation (65% and 75% increases are marked as comp− and comp+, respectively); other parameters and notations as in (*a*). (*d*) The average postsynaptic firing frequency (colour coded) over the range of *G*_*m*_ (abscissa) and *P*_*r*_ (ordinate) values, and three cases of input axon firing frequency (5, 10 and 20 Hz), as indicated; six-trial average; note different colour-coded scales.

As expected for highly nonlinear systems, such as threshold-dependent neuronal spiking, a relatively small (50%) increase in the input produced a several-fold boost of the output. However, somewhat counterintuitively, the postsynaptic effects of the equal increases in *G*_*m*_ and *P*_*r*_ were substantially different (figure 2*a*). Increasing synaptic conductance *G*_*m*_ by 50% produced a profoundly larger enhancement of the postsynaptic cell firing than increasing *P*_*r*_ by 50%: the difference amounted to several-fold at lower input frequencies (10–30 Hz) gradually disappearing at >40 Hz (figure 1*a*). Interestingly, the normalized (scale-independent) input–output transfer functions for the two scenarios (figure 1*b*) suggested that, under potentiated *P*_*r*_, the output potentiation level increases gradually with the higher input frequencies whereas the *G*_m_ increase produces a more uniform potentiation. We have also estimated that the stronger output in the case of 50% postsynaptic potentiation could be ‘compensated for’ by a 65–75% increase in *P*_*r*_ (figure 2*c*).

To assess whether such phenomena are consistent over a plausible range of *P*_*r*_ and *G*_*m*_ , we carried out similar simulation experiments while systematically changing these two parameters. The results confirmed that the postsynaptic firing output is generally more sensitive to changes in *G*_*m*_ than in *P*_*r*_, particularly at lower input frequencies (figure 2*d*; the graph gradients are consistently steeper along the *G*_*m*_ axis than along the *P*_*r*_ axis, with both axes on a similar relative scale).

**Figure 3.**
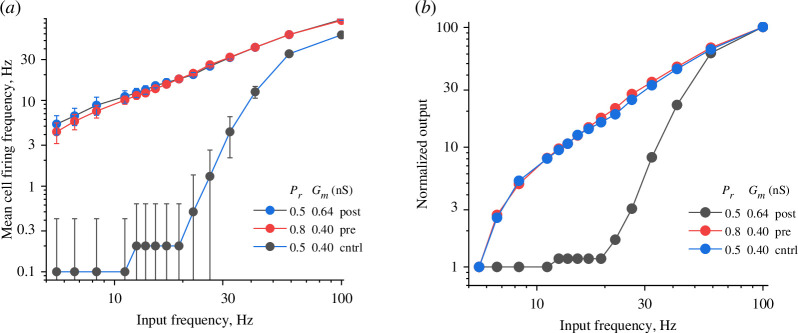
The difference between the outputs of pre- and postsynaptic potentiation collapses under high *P*_*r*_. (*a*) The frequency of postsynaptic cell firing plotted against the presynaptic afferent firing frequency, for the control conditions (cntrl) and two potentiation scenarios (pre and post), as indicated, in the simulated CA3–CA1 circuitry, under higher values of *P*_*r*_; dots, mean ± s.e.m., *n* = 10 trials; log–log scale. (*b*) The input–output dependencies are as in (*a*), but normalized to the output range of 1–100; log–log scale.

The latter data also pointed to the lessening of the firing output difference (under equal pre-and postsynaptic potentiation) with higher release probability values. We therefore carried out simulations for the *P*_*r*_ values changing by 60%, from 0.5 to 0.8 (against *G_m_* changing from 0.40 to 0.64 nS, also by 60%) and found virtually no difference between the outcomes for the two cases (figure 3). While the average *P*_*r*_ above 0.5 is unlikely to be common in the intact brain [46], these data highlight the case when equal pre- and postsynaptic changes can produce equal outcomes, which might also be affected by the short-term presynaptic plasticity (see §4).

### (b) Neural network activity under equal changes in *G*_*m*_ and *P*_*r*_

**Figure 4.**
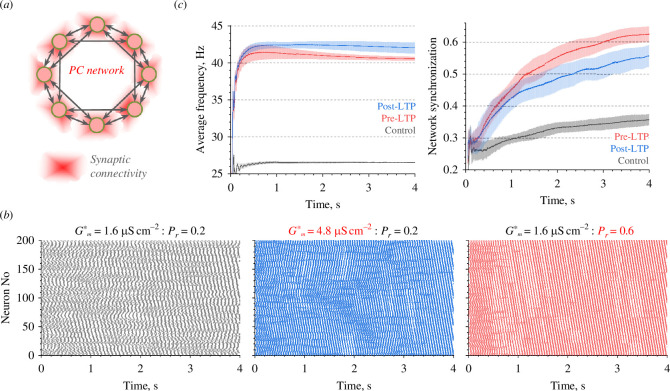
Probing the effects of pre- and postsynaptic potentiation on the basic features of neuronal network activity. (*a*) Diagram of the modelled circular network of principal neurons; red shade depicts synaptic connections clustered near individual neurons (see §2 for detail) [39–41]. (*b*) Raster plots of neuronal spiking (network of 200 cells) in control conditions (left) and under a threefold increase of either postsynaptic G*_m_ (middle) or presynaptic *P*_*r*_ (right), as indicated. (*c*) Examples, key parameters of neuronal network activity, average neuron spiking frequency (left) and network synchronization (right) evolving under control conditions and under presynaptic (pre-LTP) and postsynaptic (post-LTP) potentiation, as indicated, with the G*_m_ and *P*_*r*_ values shown in (*b*); lines and shaded areas, mean ± s.e.m. (*n* = 10 runs).

We next asked how the differential effects of pre- and postsynaptic potentiation, as seen in the modelled CA3–CA1 microcircuitry, would transpire in the activity of an interconnected neuronal ensemble. To address that, we sought to explore a generalized non-hierarchical, circular network of principal neurons with a clustered connectivity via stochastic synapses and realistic time delays [39–41] (figure 4*a*; §2).

Once initiated under the control settings, the 200-neuron network would settle in a quasi-periodic pattern of activity, as expected (example figure 4*b*, left). Next, we increased either the release probability *P*_*r*_, or the synaptic conductance density G*_m_ threefold, to reflect a wide dynamic range of these parameters in the native network-wide populations of synaptic connections [33,47,48]. Both types of change produced noticeable effects on the network activity (examples in figure 4*b*).

The quantitative analysis of these effects (§2) has indicated that the consequences of equivalent increases in G*_m_ or *P*_*r*_ for the basic features of populational neuron firing differ, too. After the initial network stabilization, the threefold increase in either G*_m_ or *P*_*r*_ elevated the average neuron firing frequency by either ~59% or ~53%, respectively (figure 4*c*, left). At the same time, the network synchronization parameter (§2) showed a somewhat opposite trend, showing increases by ~55% and ~75%, respectively (figure 4*c*, right).

## 4. Discussion

Because the two forms of LTP, presynaptic and postsynaptic, can co-exist across the brain circuits including CA3–CA1 connections [15,20–24], it seemed important to understand their comparative impact on the signal transfer, under similar changes in each of the two forms. Our simulations indicated that, in a basic synaptic microcircuit, an increase in release probability *P*_*r*_ generally produced a lesser impact than a similar increase in the synaptic receptor current *G*_*m*_. The most parsimonious explanation for this phenomenon is the nonlinearity of dendritic signal integration so that larger synaptic currents undergo partial saturation (sub-linear summation) when arriving at the soma [49,50]. An increase in *G*_*m*_ affects all local synaptic currents to the same degree, thus linearly expanding their amplitude distribution. In contrast, an increase in *P*_*r*_ boosts the numbers of larger events, thus skewing the distribution of the synaptic current amplitudes towards greater values. Because the amplitude-dependent saturation affects the larger responses to a greater degree, the increase in *P*_*r*_ biases the outcome towards a lower average compared to the effect of *G*_*m*_. The effect was particularly pronounced at reltively low input frequencies (10-25 Hz) and low *P*_*r*_ (around 0.2). We note that in such conditions, single-synapse release events occur only every 200-500 ms, suggesting little influence of use-dependent short-term presynaptic plasticity on the phenomenon in question. The outcome difference between pre- and postsynaptic potantiation gradually disappears under higher input frequencies when both potentiation forms produce predominantly large events subjected to similar sub-linear summation.

The distinct postsynaptic influences of equal increases in *G*_*m*_ and *P*_*r*_ may reflect the fact that, in real brain circuits, the dynamic ranges of these two parameters differ substantially. While the postsynaptic LTP very rarely shows the *G*_*m*_ increased above twofold, the *P*_*r*_ values could range from near-zero to near-one, even at the same cell axon [51]. Thus, the cellular systems of the brain have ample reserves to match the functional outcome of presynaptic and postsynaptic changes. On the other hand, the above disparity occurs only when the input signal frequencies are relatively low, suggesting different conditions for LTP at synaptic connections that tend to operate at higher as opposed to lower frequencies, and involving synapses with higher as opposed to lower release probability.

While the present simulation study considers no activity-dependent short-term changes in *P*_*r*_ (see above) or in postsynaptic intrinsic excitability, there is clearly an infinite number of plasticity scenarios that could be explored in the context. Neural activity may also engage highly nonlinear dendritic effects, such as dendritic spikes or NMDA receptor-driven plateau potentials, which are however more likely to occur *in vivo* when the cell experiences multiple electrogenic influences across its morphology, rather than being in the ‘resting state’ in a quiescent brain slice preparation. The present results have to be viewed therefore as proof-of-conceptrather than a generalization.

Our network simulations indicate that the effects of changing *G*_*m*_ and *P*_*r*_ on the populational activity of neurons are likely to be complex: they are not necessarily inferred directly from the effects seen in the modelled CA3–CA1 microcircuitry. The difference between the two potentiation mechanisms in the resulting network firing frequency increases was small. However, the disparity in synchronization appeared significant, probably reflecting the fact that higher *P*_*r*_ should lead to a disproportionately greater occurrence of coincident events than higher G*_m_. Clearly, the present network simulations can unveil only some basic phenomena associated with a change in *G*_*m*_ or *P*_*r*_. The expected intricacy of the effects, including the likely contribution of short-term presynaptic plasticity, suggests that it would be prudent for network exploration to focus on a specific task in a particular network setting before attempting to systematically explore the parameter space. We thus restricted ourselves here to a mere demonstration of the complexity of the case.

Nonetheless, in conditions of microcircuitry, the diverging outcomes of nominally equal pre- and postsynaptic changes in synaptic efficacy, as reported here, should help to dissect the underlying mechanism when such dissection is not directly attainable. For instance, probing the shape of a system’s input–output function (as in figure 2*b*) under various conditions could suggest a higher likelihood of one scenario over the other.

## Data Availability

The simulation platforms BRAINCELL (current version) and ARACHNE are freely available through the GitHub website [52,53]. BRAINCELL and ARACHNE can be used to obtain the simulation data as presented. The raw data represented in the illustrations can also be obtained from the authors.
